# A Novel Trial of Mirabegron and Behavioral Modification Including Pelvic Floor Exercise for Overactive Bladder in Parkinson's Disease (MAESTRO)

**DOI:** 10.7759/cureus.31818

**Published:** 2022-11-23

**Authors:** Arina Madan, Theodore Brown, Sudeshna Ray, Pinky Agarwal, Ina Roy-Faderman, Daniel Burdick

**Affiliations:** 1 General Medicine, Guy's and St Thomas' NHS Foundation Trust, London, GBR; 2 Neurology, EvergreenHealth Neuroscience Institute, EvergreenHealth Medical Center, Kirkland, USA; 3 Emergency Medicine, Eastside Research, Redmond, USA; 4 Philosophy, School of History, Philosophy, and Religion, Oregon State University, Corvallis, USA

**Keywords:** antimicrobial resistance, antimicrobial stewardship, urinary tract infection, treatment, parkinson’s disease, overactive bladder, mirabegron

## Abstract

Overactive bladder (OAB) is experienced by more than half of patients with untreated Parkinson's disease. Treatment of overactive bladder in these patients has included antimuscarinic anticholinergics, raising concerns about the possibility of exacerbating cognitive impairment or constipation. Mirabegron (Myrbetriq), a β3-receptor agonist, provides relief of OAB without increasing cognitive impairment. While prior studies have examined the effect of mirabegron on OAB in a variety of patient populations, this study is the first controlled, prospective study investigating the effect of mirabegron on overactive bladder in patients with Parkinson's disease. By studying effective treatments for overactive bladder, this trial emphasizes the importance of antimicrobial stewardship so that lower urinary tract symptoms are not treated as lower urinary tract infections with antimicrobials and instead overactive bladder can be treated appropriately with medication. The MAESTRO study compared the effect of adding mirabegron to behavioral modification (including pelvic floor exercises) to behavior modification alone. Results from this novel study show that both the mean absolute change in the volume of micturition (objective measure) and the mean percent change increased significantly between visits two and three in the experimental group using mirabegron. Moreover, improvements in micturition in this study indicate that a larger-scale study of mirabegron with pelvic floor exercises and behavior modification is warranted.

## Introduction

Parkinson’s disease (PD) is a neurological disease characterized by multi-system degeneration with progressively disordered motor and non-motor symptoms. While the clinical diagnosis is based on the emergence of motor signs and symptoms, such as bradykinesia, rigidity, and resting tremor, progression typically results in non-motor symptoms including neuropsychiatric symptoms like dementia and psychosis, sleep disturbances like insomnia and daytime somnolence, and autonomic dysfunction like orthostatic hypotension and bladder and bowel dysfunction.

Overactive bladder (OAB) is experienced by as many as 65% of patients with untreated PD [1]. OAB is characterized by urinary urgency, usually accompanied by increased frequency of daytime urination and nocturia, with no other known cause. Often PD patients are inappropriately treated with antibiotics for lower urinary tract infections instead of receiving the correct treatment for overactive bladder. This study has a strong emphasis on improving antimicrobial stewardship by developing effective and appropriate treatment for overactive bladder in PD patients.

For many years, the principal pharmacotherapy options for management of OAB were antimuscarinic anticholinergics, such as oxybutynin, tolterodine, and solifenacin. Further, patients with PD are especially sensitive to cognitive side effects of anticholinergic medications because they, by nature of their disease, have a significant cholinergic deficit [2]. In addition, PD patients have a high rate of constipation, another symptom that may be significantly worsened by anticholinergic medications [3]. Moreover, use of anticholinergics in the general population appears to increase the future risk of dementia, a risk that is already increased in PD patients. Two commonly used anticholinergic medications, oxybutynin and tolterodine, were consistently associated with dementia after long-term (>4 years) use in elderly patients, even when exposure was 15-20 years prior to the onset of dementia [4]. Anticholinergic burden in elderly persons results in cognitive decline, episodes of delirium, and subsequent need for emergency care. In addition, the use of some antimuscarinic agents is associated with an increased risk of developing or worsening dementia [5,6].

The combination of potential side effects of anticholinergic agents suggests that identifying effective alternatives for treating OAB in PD patients is desirable. When approved for use in the United States by the US Food and Drug Administration (FDA) in 2012, the β3-adrenoceptor agonist mirabegron offered the possibility of pharmacological management of OAB without the risks associated with anticholinergic agents. Because mirabegron avoids anticholinergic effects such as constipation and possible cognitive impairment and decline, it would be expected to be better tolerated by patients with PD [7].

In clinical trials, mirabegron has been shown to be efficacious in the treatment of OAB in the general adult population. A phase 3, double-blind, parallel-group, placebo- and tolterodine-controlled study of mirabegron in adult patients with OAB showed statistically significant improvements over placebo at 50 mg and 100 mg daily, particularly in decreasing episodes of incontinence and number of micturitions in a 24-hour period [8].

The clinical trials that led to the approval of mirabegron excluded patients with neurological conditions including Parkinson’s disease. A few recent, open clinical trials suggest that mirabegron may be effective in controlling bladder dysfunction specifically in patients with PD. In a 2017 retrospective study, 19 patients who had intolerable side effects from anticholinergic medications used to treat OAB were given mirabegron (50 mg) over the course of six months; of the 13 patients who stayed in the study, both urinary urgency and urgency-related incontinence decreased [9]. A later retrospective study of mirabegron for the treatment of OAB in PD patients showed improvements in the OAB symptoms of 50% of the patients which persisted in over half of the patients who showed improvement; moreover, this study showed low rates of adverse events (AEs) [10]. A 30-patient, open-label clinical trial tested safety and efficacy of mirabegron in patients with PD who had OAB that was non-responsive to other categories of medications. Of the patients who completed the study, nearly one-quarter (23.3%) achieved complete urinary continence. Statistically significant decreases in the daily frequency of urinary urgency and urge urinary incontinence (UUI) episodes were observed in 24 patients [11,12].

Pelvic floor exercises (PFE) and other behavioral modifications have been explored as a method of improving bladder control and function in patients with Parkinson’s disease. A recent, randomized study showed improvement in OAB symptoms and quality of life for PD patients who participated in PFE and other behavioral modifications relative to patients in the control group, though episodes of incontinence were reduced in both groups. An open pilot trial of 20 patients with PD showed significant improvement in episodes of urinary incontinence from baseline to follow-up after an eight-week, five-visit course of PFE-based behavioral therapy [13].

As both the early mirabegron trials and the PFE studies were open-label, single-armed, and/or retrospective studies, the MAESTRO study provides crucial prospective, double-blind, placebo-controlled information on the assessment of mirabegron with PFE as a treatment combination for OAB in PD patients. Moreover, this study provides useful information for development of a larger study of the potential use of mirabegron in these patients.

A preliminary report on this MAESTRO pilot study was previously published [14]. Previous trials were not double-blind and, therefore, we are doing a randomized, double-blind trial to further evaluate the use of mirabegron and pelvic floor exercises in overactive bladder in Parkinson’s disease patients.

## Materials and methods

This paper presents study design considerations and results of the 10-week MAESTRO study. In contrast to prior studies of OAB and mirabegron use as described above. The MAESTRO study was a prospective, randomized, double-blind, placebo-controlled trial of the effectiveness of mirabegron as an add-on therapy to an educational intervention of behavioral modification including pelvic floor exercises (PFE) on OAB in PD patients. Specifically, the study compared the effectiveness of FDA-approved dosage strengths of mirabegron combined with behavioral modifications (including PFE) in improving bladder control and function in patients with PD with the effect on OAB of PFE alone.

Organization

The clinical trial was performed at the Booth Gardner Parkinson's Care Center in Kirkland, Washington, and supported by an unrestricted research grant from Astellas Pharma Inc., manufacturer of Myrbetriq® (mirabegron). Astellas Pharma, based in Tokyo, Japan provided both medication and comparable placebo. Astellas Pharma had no input on trial design, trial performance, data analysis, or reporting of outcomes. The protocol and consent procedures and forms were approved by the Western Institutional Review Board. The institutional review board (IRB) also monitored the safety, data integrity, and progress of the trial. Western Institutional Review Board issued approval #1145312 for this study and the clinical trial number is NCT02092181.

Participants

Interested patients were identified when receiving clinical care at the EvergreenHealth Neuroscience Institute or by referral from regional clinical organizations. Before participating in initial screening procedures, patients were provided with information about the trial, and a signed and dated IRB-approved informed consent form was required before any protocol-specific screening procedures were performed.

Patients were screened for participation during their first visit (from here identified as Visit 1) on the basis of Parkinson’s disease symptoms, lower urinary tract symptoms (LUTS), medical history, PD treatment history, and treatment history of OAB. To be eligible for participation, the patient must have had a confirmed diagnosis of PD, stable PD medication for at least four weeks prior to Visit 1, and no use of anticholinergic bladder medications for at least two weeks prior to Visit 1. However, patients could be enrolled if they received pro re nata (PRN) doses of carbidopa-levodopa to address periodic worsening of Parkinsonian symptoms. Criteria for inclusion and exclusion from participation are shown in Tables 1, 2.

**Table 1:** Study participation inclusion criteria for trial participation. *Participation also requires that doses do not change during the study, except PRN doses of carbidopa/levodopa will be allowed to address periodic worsening of parkinsonian symptoms. PD: Parkinson's disease; UTI: urinary tract infection

Inclusion criteria	
Aged 30+ years	
Diagnosis of PD consistent with UK PD Society Brain Bank criteria	
No change in PD medications during four weeks preceding screening*	
Willing and able to complete micturition diary	
Experiences urinary urgency: having eight or more entries of bladder urgency score >2 in 72-hr voiding diary during screening period	
Micturition frequency, defined by	8+ episodes of micturition per 24 h, averaged over 72 h; or
	2+ episodes of incontinence in 72 h, documented in voiding diary during screening period
May use other medications that could influence bladder function, as long as dose is stable for four weeks preceding screening, with no dose changes during the study	
Valid health insurance coverage at the time of study enrollment. expects coverage to remain valid for duration of study period	

**Table 2:** Study participation exclusion criteria for trial participation. PD: Parkinson's disease; UTI: urinary tract infection; AST: aspartate aminotransferase; ALT: alanine transaminase

Exclusion criteria		
Woman who is breast-feeding, pregnant, or has potential to become pregnant during the course of study		
Cognitive deficits that, per study physician, would interfere with patient’s ability to give informed consent or perform study testing		
Evidence of chronic inflammation, e.g., interstitial cystitis, bladder stones, previous pelvic radiation therapy		
Previous or current malignant disease of the pelvic organs		
Intravesical botulinum toxin treatment within six months prior to screening		
InterStim device		
Indwelling catheter or self-catheterization		
Concurrent use of thioridazine, flecainide, propafenone, digoxin, or warfarin		
Use of anticholinergic bladder medications specified within 14 days of screening (patients who used one of these and discontinued 14+ days prior to screening are not excluded)		Darifenacin (Enablex), fesoterodine (Toviaz), flavoxate (Urispas)
		Oxybutynin (Ditropan, Ditropan XL, oxetol, Uromax, Apo-Oxybutynin, Riva-Oxybutynin, Cystrin)
		Solifenacin (VESIcare)
		Tolterodine (Detrol, Detrol LA, Detrusitol, Unidet)
		Trospium (Sanctura, Sanctura XR)
An estimated glomerular filtration rate (eGFR) <60, or AST or ALT >2x upper limit of normal		
Other serious and/or unstable medical conditions		
Participated in other drug studies or has used other investigational drugs within 30 days of screening		
At screening	Systolic blood pressure >165 or diastolic blood pressure >100	
	Heart rate >100 beats per minute at screening	
	History of Mirabegron allergy	
	Post-void residual >200mL	
	Evidence of UTI	

Of the 53 patients who were screened for participation, approximately one-third (19 patients) did not meet medical criteria for participation which included: urinary tract infection (UTI, five patients), insufficient voiding diary reports of incontinence (four patients), low glomerular filtration rate (GFR) (four patients, one with high creatinine), excessive post-void residual volume (three patients), other illness (two patients, myeloma, prostate cancer), and Parkinson’s medication changed within four weeks of evaluation (one patient). The other four patients who were screened chose not to enroll in the study. At the time of study initiation, efficacy data regarding the use of mirabegron in this patient population were not available, so calculations of sample size needed to power such a study could not be performed. Given the limited number of patients who met inclusion criteria, the study enrolled 30 candidates who met these criteria and agreed to trial participation.

Study design

**Figure 1:**
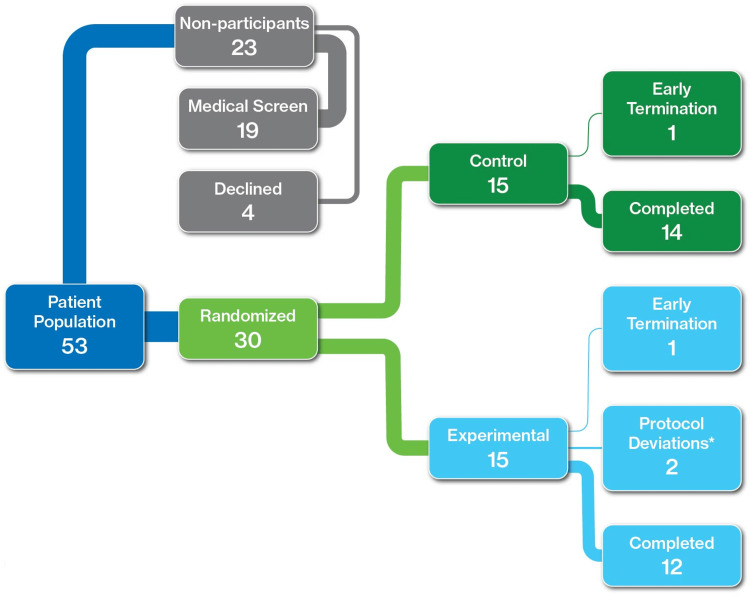
Study design including randomization, non-participants, experimental, and control groups. *Each patient missed three days of monitoring and recording diaries, one during Visit 3 window and one during Visit 4 window.

This investigator-initiated study was a placebo-controlled, double-blind, randomized, prospective, and single-site trial. Subjects were enrolled in the trial based on responses to an overactive bladder questionnaire at baseline (Visit 2). Over the course of 10 weeks, a total of 30 eligible PD patients over the age of 30 with overactive bladder (OAB) were randomized into control or experimental groups (Figure 1).

The study comprised four clinic visits and two phone visits over the course of 12 weeks, with bladder function and related qualitative assessments performed over the course of the study (as described in the assessments section below). The control group received an educational intervention of behavioral modification including pelvic floor exercise (PFE). The behavioral modification program was training-based. The intervention included a 15-20 minute presentation created by a physical therapist, with slides providing information on bladder habits, bladder anatomy, bladder retraining, and urge-delay techniques. The presentation also included instructions on performing the Kegel exercises the patients would be practicing, integrating these exercises into daily activities, and maintaining the PFE program. Patients had the opportunity to ask the study physician follow-up questions and received a paper copy of the presentation to take home for reference. Compliance was assessed using the subject's bladder diary - they were asked to record the date, time of day, and duration of exercise every time they performed the Kegel exercises.

The treatment group received the above training as well as mirabegron as add-on therapy. This group received 25 mg of mirabegron daily with a planned titration to 50 mg daily with the option to not increase the dose at Visit 3 if OAB symptoms were fully controlled by the lower dose or due to limited tolerability. Study drug accountability and reconciliation were performed to ensure adherence to the medication regimen.

Intervention

**Figure 2:**
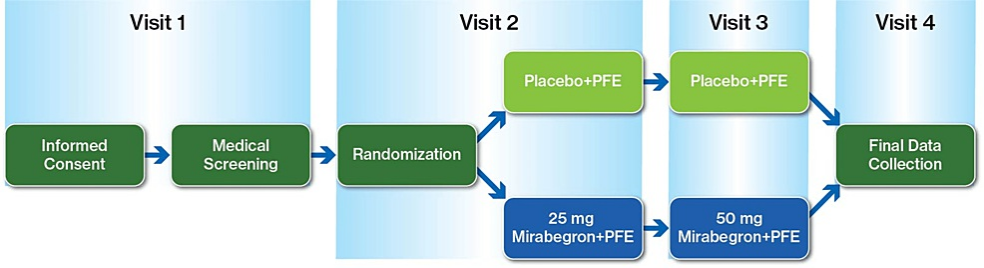
Study design: Visit 1 - medical screening, Visit 2 - randomization, Visit 3 - PFE vs PFE+mirabegron, Visit 4: final data collection. PFE: pelvic floor exercises

The group of 30 screened patients with PD was divided into two groups using a computer-generated 1:1 randomization scheme, with the research pharmacist retaining sole access to the code book of treatment allocation (with exceptions made in cases of medical emergency, of which none were reported). The control group received behavioral modification training and participated in regular pelvic floor exercises (PFE) followed by placebo manufactured and provided by Astellas Pharma. The experimental group added mirabegron treatment to the PFE regimen (Figure 2). Patients in the experimental group received the initial dosage of mirabegron, 25 mg daily (prescribing information, Astellas Pharma, Inc.). A higher dose of 50 mg daily is the highest dose approved by the FDA for use in patients with OAB, so the option to titrate to 50 mg at the mid-point of the trial was provided for patients who did not experience significant side effects and might have derived additional benefit from an increased dose of mirabegron.

Efficacy assessment

The primary outcome measure of the effect of the study treatment (mirabegron in combination with PFE) was a change in the overactive bladder-symptom composite score (OAB-SCS) between each patient’s baseline score (determined during Visit 2) and the score on Visit 4 [15]. The pre-specified secondary outcomes measured between visits include the OAB-SCS, which includes a self-assessment of symptom severity as well as an assessment of quality of life impact (OAB-q) between patient baseline and Visit 3 [15]. Other secondary outcomes include - mean number of micturitions in a 24-h period [16], mean volume (mL) per micturition [16], mean number of urinary incontinence episodes per 24 h [16], overactive bladder questionnaire (OAB-q) score [17], Subject Global Impression of Change (SGI-C) score [18], Patient Perception of Bladder Condition (PPBC) score [19], Urgency Scores (also incorporated into OAB-SCS) [15], and Non-Motor Symptoms Scale (NMSS) scores [15-20]. The NMSS score includes questions about cognition, psychosis, and constipation, in addition to urinary symptoms [20].

The measurement and assessment tools used for secondary outcomes are provided in Table 2. In addition to the change between the baseline overactive bladder-symptom composite score and the score on Visit 3, secondary outcome measures were chosen to identify changes in bladder function and control. These variables were recorded at each clinic visit or in voiding diaries kept by patients between visits. The following functional measurements were based on patient voiding diaries: mean number of micturitions per 24 h, mean number of incontinence episodes per 24 h, and mean volume voided per micturition. Patients were provided with these assessments for completion during clinic visits: overactive bladder questionnaire, Non-Motor Symptoms Scale (NMSS), Patient Perception of Bladder Condition (PPBC), and Subject’s Global Impression of Change (SGI-C) [15-20].

Statistical analyses

Absolute changes in mean outcome measures of the control and treatment groups were compared. In addition, percent changes relative to baseline were determined for each measure, noting any trends indicated by differences between the two groups. Since individual patients in control and test groups were not paired, standard two-tailed T-tests were chosen to be the most appropriate option for determining the significance of any notable changes. Since variance differed in control and treatment groups, mixed-variance T-testing was used.

Safety assessment

Safety assessment involved monitoring and recording all adverse events (AEs) as well as categorizing each event as mild, moderate, or severe, defined as follows:* *mild event causes transient or minimal symptoms and does not interfere with daily activities, moderate event causes symptoms sufficient to interfere with but not prevent daily activities, severe event that prevents normal, everyday activities. Patients were monitored for AEs throughout the course of the study through phone visits, during clinical Visits 3 and 4, and continuously through phone contact and unscheduled visits as needed.

Any of the following was considered an AE: adverse drug reactions, onset of illness during the study, exacerbations of pre-existing conditions, clinically significant changes on physical examination, and significantly abnormal objective test findings. Worsening of a medical condition or a disease that was present before the initiation of the study treatment was also considered an adverse event. Post-void residual urine volume was assessed at screening, Visit 3, and Visit 4 as an indicator of efficacy of the trial protocol and to screen for urinary retention as an adverse event. Subjects who discontinued the study medication due to AEs were followed until the adverse event or events ended. Adverse events received continuous scrutiny during the observations. The rate of such events was comparable to AE rates that have been recorded in published PD drug trials.

## Results

Study population

Thirty patients were entered into the study. All patients included were over the age of 30 with a diagnosis of Parkinson’s disease (UK Brain Bank Criteria) [21]. Patient demographics are provided in Table 3. Baseline characteristics including baseline assessments of OAB of the 30 patients entered into the study are provided in Table 4.

**Table 3:** Patient demographics including age, sex, weight, and years since Parkinson's disease diagnosis. PFE: pelvic floor exercises; PD: Parkinson's disease

Variables	PFE only	PFE+mirabegron
Male	66.7%	80.0%
Female	33.3%	20.0%
Age (years), mean (range)	67 (60-81)	66 (53-85)
Weight (lbs.), mean (range)	179.1 (108-308)	183.5 (113-255)
Years post-PD diagnosis (range)	6.2 (1-21)	4.7 (0.5-12)

**Table 4:** Patient baseline characteristics including disease duration and scoring systems for overactive bladder symptoms. MDS-UPDRS: Movement Disorder Society-Unified Parkinson’s Disease Rating Scale; OAB-SCS: overactive bladder-symptom composite score; OAB-q: overactive bladder questionnaire; NMSS: Non-Motor Symptoms Scale Scores

Baseline (Visit 2)	PFE only	PFE+mirabegron
Disease duration (years)	5.8	4.7
Hoehn-Yahr (HY) Stage	2.3	2.1
MDS-UPDRS Motor 3 Score	33.4	30.9
Mean daily OAB-SCS (mean of micturitions per 24-h period)	28.1	23.4
OAB-q	29.1	23.4
NMSS (baseline)	74.5	58

Efficacy

Primary Endpoint

The primary endpoint for this study was the change in OAB-SCS between Visit 2 (baseline) and Visit 4 (on maximally tolerated study drug dose - 25 mg or 50 mg - or matching placebo). The primary endpoint for this study was not achieved. The difference in the average Visit 4 OAB-SC scores in the experimental and control groups was not statistically significant nor was the difference between baseline and Visit 4 within either of the groups (Table 5).

**Table 5:** Primary endpoint: change in OAB-SCS between Visit 2 (baseline) and Visit 4 (on maximally tolerated study drug dose - 25 mg or 50 mg - or matching placebo). OAB-SCS: overactive bladder-symptom composite score; PFE: pelvic floor exercises

Variables	Control (PFE)	Experimental (PFE+mirabegron)	p-Value
Mean OAB-SCS at baseline	28.1	23.4	-
Mean OAB-SCS at Visit 4	19.2	20.2	-
Δ Mean OAB-SCS	-8.9	-3.21	0.079 (not significant)
Mean % Δ	-31.4%	-13.7%	0.144 (not significant)

Secondary Endpoints

The change in OAB-SCS between baseline and Visit 3 (25 mg of study drug or matching placebo) was a pre-specified secondary endpoint. The other secondary endpoints (also pre-specified) were analyzed for changes between baseline and Visit 3, and baseline and Visit 4 (Table 6).

**Table 6:** Secondary endpoints: baseline and post-baseline: OAB-SCS, NMSS, OAB-q, PPBC, S-GIC, micturitions, volume, and incontinence per 24 h. *Mean scores, baseline and post-baseline. **Baseline for measures other than S-GIC obtained on Visit 2. S-GIC baseline obtained on Visit 3 per 24 h period, per urination. ***Statistically significant difference compared with the control group. V: visit; OAB-SCS: overactive bladder-symptom composite score; NMSS: Non-Motor Symptoms Scale Scores; OAB-q: overactive bladder questionnaire; PPBC: Patient Perception of Bladder Condition; S-GIC: Subject’s Global Impression of Change

Endpoint*, Visit number	Control PFE-only				Experimental PFE+mirabegron			
Primary	Baseline**	Post-baseline	Δ	%Δ	Baseline**	Post-baseline	Δ	%Δ
OAB-SCS, V4	28.1	19.2	-8.9	-30.1%	23.4	20.2	-3.2	-13.7%
Secondary	Baseline**	Post-baseline	Δ	%Δ	Baseline**	Post-baseline	Δ	%Δ
OAB-SCS, V3	29.0	22.2	-6.8	-21.1%	23.4	17.3	-6.1	-25.1%
NMSS, V3	74.5	56.5	-19.6	-26.4%	58	50.5	-7.5	-14.2
NMSS, V4	74.5	56.6	-17.8	-26.0%	58	47.5	-10.5	-16.4
OAB-q, V3	27. 9	24.6	-3.2	-10.1	22.5	18.7	-4.1	-18.5
OAB-q, V4	27. 9	20.7	-8.6	-20.4	22.5	20.2	-2.6	-9.7
PPBC, V3	4.2	3.4	-0.7	-16.4	3.4	3.3	-0.4	13.2
PPBC, V4	4.2	3.2	-1.2	-26.2	3.4	3.1	-0.4	9.02
Micturations (per 24 h), V3	9.8	8.1	-1.7	-16.8	8.9	8.0	-0.9	-9.1
Micturations (per 24 h). V4	9.8	7.9	-1.9	-18.1	8.9	7.9	-1.0	-10.7
Volume (per urination) mL, V3	178.9	165.4	-13.6	-5.0	191.06	220.9	29.9***	15.6***
Volume (per urination) mL, V4	178.9	172.2	-4.9	0.1	191.06	184.7	-8.0	-2.3
Incontinence (per 24 h), V3	1.8	1.7	-0.1	-6.0	0.7	0.6	-0.1	-8.5
Incontinence (per 24 h), V4	1.8	1.1	-0.7	-37.6	0.7	0.8	0.1	23.1
Urgency, V3	3.0	2.8	-0.2	-5.3	2.6	2.4	-0.2	-7.3
Urgency, V4	3.0	2.6	-0.7	-22.2	2.6	2.2	-0.3	-10.9
S-GIC** V3, V4	3.1	2.6	-0.5	-2.9	3.5	3.5	0	-

Both the mean absolute change in the volume of micturition (objective measure) and the mean percent change increased significantly between Visit 2 and Visit 3 in the experimental group. In contrast, the control group’s mean volume of micturition decreased between baseline and Visit 3. The differences in the experimental group were statistically significant (volume change, p=0.032; %volume change, p=0.026). By Visit 4, the mean volume of micturition in the control group had returned to near baseline; in contrast, the mean for the experimental group showed no statistically significant change between Visits 3 and 4. There was no significant difference between the control and treatment groups in the other secondary endpoints, nor were significant changes identified between baseline and Visits 3 or 4 in these endpoints for the treatment group.

Safety

While more control group subjects suffered severe adverse events (AEs) than experimental group subjects, more patients total in the experimental group suffered adverse events (mild, moderate, and severe) than those in the control group. One patient in each group discontinued participation due to adverse events - psychosis in the control group and abdominal cramping in the active group. One person in the experimental group experienced an increase in PD symptoms.

Three moderate AEs occurred within the control group. One patient suffered psychosis and the study medications were discontinued for this patient. Another patient suffered cellulitis, facial abscess, and pneumonia. The patients in the experimental group reported no moderate AEs.

Patients in both study groups experienced mild adverse events. The experimental group had more AEs which were reported by two or more individuals - hallucinations, fatigue, and edema of lower extremities. Many non-mild and moderate AEs were experienced by single subjects in one of the two groups. These events included dyskinesia, sinus infection, hypertension, knee pain, and hallucination. Two mild AEs were experienced by two individuals - cold symptoms and falls. Both individuals were members of the control group.

Several types of adverse events, however, were notable for being experienced by more than one patient in each group (Table 7). Adverse GI events were reported by patients in both groups. Six patients in the experimental group reported diarrhea, loose stools, or constipation (with resultant hemorrhoids). In addition, one patient reported abdominal cramping. Only one patient in the control group reported a GI-related adverse event, diarrhea. None of the adverse events were life-threatening and no participants required hospitalization as a result of adverse effects.

**Table 7:** Number of patients experiencing different adverse events. *Non-severe mild and moderate adverse events (AEs).

Adverse event type	Control (number of patients)	Experimental (number of patients)
Patients experiencing severe AEs	2	0
Psychosis	1	-
Cellulitis (with abscess or other sequelae)	1	-
Pneumonia	1	-
Patients experiencing non-severe AEs*	6	9
Extreme dyskinesia	1	-
Cold symptoms	2	-
Falls	2	-
Hypertension	-	1
Fatigue	-	2
Hallucinations	-	1
Dizziness	-	1
Itchy/swollen hands	1	-
Sinus infection	-	1
Foot or knee pain	1	1
Erectile dysfunction	-	1
Edema	-	1
Headache	-	1
Sore throat	-	1
Heart palpitations	-	1
Urinary incontinence	-	2
GI events	2	8
Diarrhea	1	1
Loose stools	-	2
Constipation	-	3
Abdominal cramps	-	1
Dysphagia	-	1
Heartburn	1	-

Efficacy

This novel study of 30 patients with Parkinson’s disease demonstrated potential benefits of the use of mirabegron in conjunction with pelvic floor exercises and other behavioral modifications for the management of OAB and is the first prospective, randomized, placebo-controlled trial to do so. The study did not achieve significance in its primary endpoint, change in the average overactive bladder-symptom composite score, between baseline (Visit 2) and Visit 4.

Statistically significant changes in the mean volume of micturition were seen in the treatment group but not in the control group. The percentage of patients who experienced improvements in volume per micturition was higher in the experimental group than in the control group.

The treatment impact on volume per micturition is consistent with the results from other studies which show that mirabegron increases volume voided (and thus reduces volume of urine retained after micturition) and also that mirabegron has a lower side effect profile than anticholinergics which are associated with many cognitive side effects [22,23]. In the pre-approval BLOSSOM study, for example, mean volume voided increased in the mirabegron treatment group with a dosage of 150 mg (a higher dose than was later approved by the FDA for use), in contrast to the placebo group in which no significant volume increase took place [24]. Similarly, mean voided volume per micturition increased significantly when patients received doses greater than 50 mg of mirabegron in the DRAGON study [25]. However, in the MAESTRO study, the increase was seen at Visit 3 but not maintained through Visit 4, suggesting the absence of a dose effect or that the effect was not maintained.

A few small-scale studies have suggested that PFE alone (without medication) may reduce urinary incontinence in patients with PD [13]. Bladder function in the MAESTRO study population was not affected as noticeably by PFE alone (significantly improving two measurements of bladder function in the control group - number of incontinence episodes and NMSS score). The differences seen between patients in other studies and this study may be related to baseline health and functioning of the patients. Patients enrolled in the MAESTRO study may have had more significant barriers to improvement of OAB, and thus may have been affected less by the addition of mirabegron than patients with less intractable bladder control issues.

Since many previous studies showing the efficacy of mirabegron in OAB included broader, less-delimited patient populations than were included in the MAESTRO study, a study of mirabegron and PFE with a larger sample of PD patients may be helpful in establishing whether some PD patients with OAB would experience an improvement in OAB using this combination approach to treatment. Table 8 shows assessment efficacy and load.

**Table 8:** Assessment efficacy (usability, patient and staff burden, effectiveness) and load. OAB-SCS: overactive bladder-symptom composite score; OAB-q: overactive bladder questionnaire; S-GIC: Subject’s Global Impression of Change; PPBC: Patient Perception of Bladder Condition; NMSS: Non-Motor Symptoms Scale Scores

Tool	Usability	Patient burden	Effectiveness	Staff burden
Urgency scores (OAB-SCS)	Moderate	Coaching required (reminders to complete diary) Recall of urination episode	Real-time, specific	Transcription from paper diary to database
Voiding diary	Moderate	Coaching required (reminders to complete diary)	Real-time, specific	-
OAB-q	Easy	<2 minutes to complete, requires recall	-	-
SGI-C	Easy	<2 minutes to complete, requires recall	-	-
PPBC	Very easy	-	Non-specific, little useful information	-
NMSS	Moderate	15 minutes time	Not effective Added time recording non-urinary symptoms	Adds 15 minutes with patient for ratings

Safety and tolerability

In the study population, serious adverse events were limited to the control group (20% of patients in control group) and infrequent (9.6% of the total enrolled population). No treatment-related AE trends were observed.

CNS side effects of any medication must be considered for patients with PD, who already experience cognitive impairment. The possibility for increased CNS impairment is a particular concern for anticholinergic OAB medications, many of which were approved and used prior to the development and approval of mirabegron. Patients with PD are sensitive to cognitive side effects of many anticholinergic agents because they, by the nature of their disease, have an existent cholinergic deficit. Such agents can inhibit muscarinic acetylcholine receptors (mAChRs) in the bladder detrusor muscle and inhibit voiding contraction, however, these drugs can also impact cognitive function. Studies of anticholinergic agents used to treat OAB show a range of effects on cognitive function. Older agents, oxybutynin, and tolterodine seem to have little receptor subtype specificity and, relatedly, more significant CNS effects. Oxybutynin, which can cross the blood-brain barrier, causes impairments including lowered reaction time, pattern memory, and five other cognitive tests, as well as headache, somnolence, and anxiety [26,27]. While tolterodine has fewer effects, patients on tolterodine show decline in three cognitive tests as well as headache and somnolence [28,29]. Newer antimuscarinic agents approved for the treatment of OAB, darifenacin, solifenacin, and trospium chloride, have more limited, though still noticeable, CNS effects [30]. In addition, a concern remains that the long-term use of anticholinergic medications may increase the likelihood of dementia [31,32].

Given the mechanism of action of mirabegron, fewer CNS effects would be expected compared with anticholinergic OAB treatments [33]. Mirabegron is a β3-adrenergic receptor antagonist and is of particular interest for use for OAB in the PD population because its mechanism of action may not impact CNS function as significantly as anticholinergic medications. In a placebo-controlled study of more than 800 patients aged 75 or older, mirabegron did not significantly affect cognition, in healthy patients or patients with pre-existing cognitive impairments [34,35].

The high proportion of adverse events experienced by patients in the control arm of the trial suggests that the underlying health issues of the patient population being studied may make identification of treatment-related side effects more difficult. In addition, it is unclear whether other adverse effects (e.g., changes to cardiac function which have been seen in other trials of mirabegron) would appear if patients remained on the drug for longer. In addition, some pre-approval studies of mirabegron included testing higher doses of mirabegron; fewer adverse events may have occurred in the MAESTRO study because the doses were lower, within the range approved by the FDA.

## Discussion

For patients with Parkinson’s disease, overactive bladder has a significant impact on their quality of life [17]. The benefits of pharmacological management must therefore be balanced with negative effects of such interventions on patient function and comfort. Particular attention must be paid to the impact on cognitive function in older patients, given the evidence of association of anticholinergic medications, sometimes used to treat OAB, with cognitive impairment in older persons [22]. Hence, the importance of looking at alternative options such as mirabegron and pelvic floor exercises, which is what the MAESTRO study does.

In clinical trials, mirabegron has been shown to be efficacious in the treatment of OAB in the general adult population, a treatment needed due to the cognitive side effects associated with anticholinergics [2-6]. One double-blind study of OAB showed statistically significant improvements over placebo at 50 mg and 100 mg daily of mirabegron, particularly in decreasing episodes of incontinence and number of micturitions in a 24-h period [8].

However, the clinical trials that initially led to the approval of mirabegron excluded patients with neurological conditions including Parkinson’s disease. Since then, there have been a few recent, open clinical trials suggesting that mirabegron may be effective in reducing lower urinary tract symptoms in PD patients and with a low adverse event profile [10-12]. There have also been studies of mirabegron and pelvic floor exercises showing a reduction in lower urinary tract symptoms with one study showing improvements in LUTS after only a five-visit course of PFE-based behavioral therapy [13]. Further, there are limitation with the early mirabegron studies and pelvic floor exercise studies including the fact that they were opened labeled, single-armed, and some retrospective.

This makes the MAESTRO study crucial as it provides prospective, double-blind, placebo-controlled information on the assessment of mirabegron with PFE as a treatment combination for OAB in PD patients. It also provides useful information for the development of a larger study of the potential use of mirabegron in these patients.

The MAESTRO study compared the effects of mirabegron and pelvic floor exercises with the effect of pelvic floor exercises alone on OAB in patients with PD. The MAESTRO study differs from previous studies of the efficacy of mirabegron for OAB in being placebo-controlled, prospective, and including a comparison between behavioral modification with medication and behavioral modification alone. The study identified one significant effect on OAB in the treatment arm of the study.

The MAESTRO study has some limitations. The number of patients in the study was 30 so a study with a larger number of patients would be more representative of the general population of PD patients. In developing a larger study of the impact of combining mirabegron and PFE for the treatment of OAB in patients with PD, this novel study provides information that may be useful in conducting a larger-scale trial, as well as insight into the effective use of assessment tools for this patient population.

In addition, many potential subjects for the MAESTRO trial were eliminated from participation because they were on bladder medications that rendered them ineligible for the study. For a larger study, patients on such medications could be included, provided they had been on a stable dose for at least four weeks and were otherwise eligible for the study based on bladder symptoms.

In the MAESTRO study, while significant improvement in micturition volume indicates that the combination of mirabegron and behavior modification may improve bladder function in patients with Parkinson's disease to a greater extent than behavior modification alone, a larger study may be required to determine whether there is a broader effect of mirabegron when combined with PFE on OAB in PD patients.

## Conclusions

This novel study of 30 patients with Parkinson’s disease demonstrated potential benefits of the use of mirabegron in conjunction with pelvic floor exercises and other behavioral modifications for the management of OAB and is the first prospective, randomized, placebo-controlled trial to do so. The limited number of serious adverse effects in the treatment arm of the study indicates mirabegron may be better tolerated than anticholinergics in PD patients with OAB.

Importantly, statistically significant changes in the mean volume of micturition were seen only in the treatment group. The percentage of patients who experienced improvements in volume per micturition was higher in the experimental group. The study did not achieve significance in its primary endpoint, change in the average overactive bladder-symptom composite score, between baseline (Visit 2) and Visit 4 or other secondary endpoints. This study is the first prospective, randomized, placebo-controlled trial to show promising results of using mirabegron in combination with pelvic floor exercises for the treatment of OAB in PD patients. A study of mirabegron and PFE with a larger sample of PD patients would be helpful in supporting this.
